# Supplementary material for: The spatial proteome of the Plasmodium falciparum schizont illuminates the composition and evolutionary trajectories of its organelles
Source: Nat Commun. 2026 May 30;17:6192. doi: 10.1038/s41467-026-73664-2 (PMC13369866; doi:10.1038/s41467-026-73664-2)
Supplement: Supplementary file 1 — Supplementary Information [file 41467_2026_73664_MOESM1_ESM.pdf]

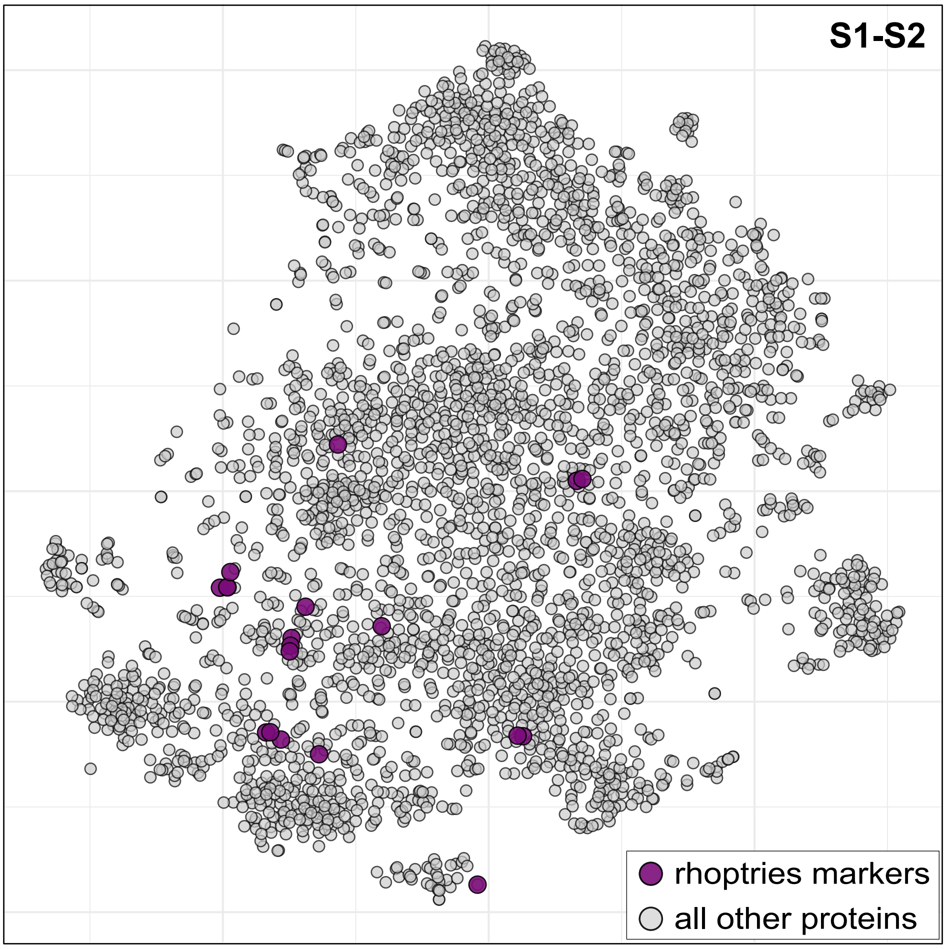

Figure S1. Rhoptry markers do not cluster in the S1-S2 combined analysis
t-Distributed Stochastic Neighbor Embedding (t-SNE) projection of 3000 proteins from the S1-S2 concatenated data with rhoptry marker proteins shown in purple.

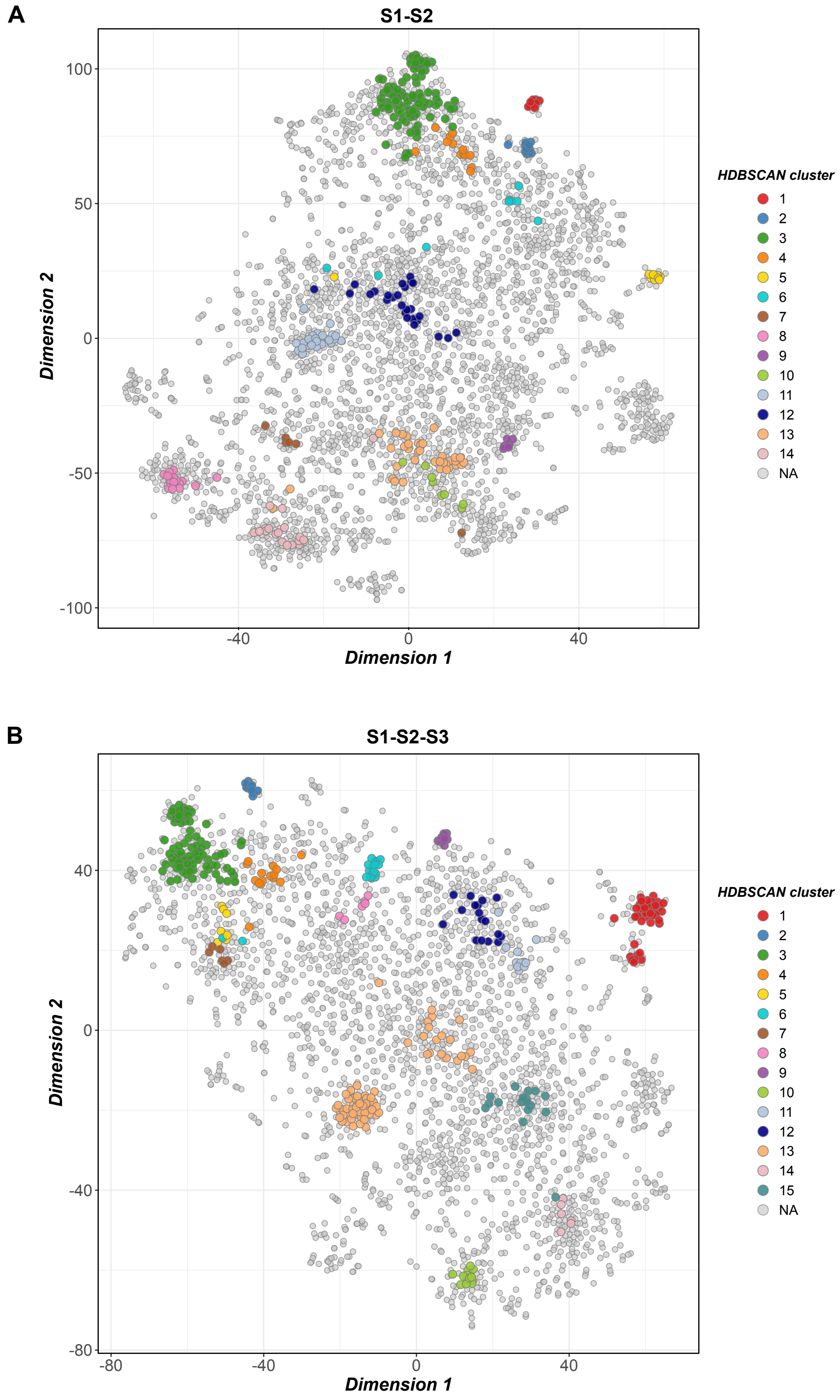

Figure S2. Unsupervised clustering using HDBSCAN
Protein clusters discovered by analysis of raw abundance-distribution profiles of the S1-S2 dataset (A) and S1-S2-S3 dataset (B) with Hierarchical Density-Based Spatial Clustering of Applications with Noise (HDBSCAN) overlaid on the t-SNE projections. Distinct clusters are numbered and indicated by colour. Core distance was set to the 8th nearest neighbour (*min_samples* = *8*) with a minimum cluster size of 8 (*min_cluster_size* = *8*). Clusters were defined using the 'leaf' method; all other parameters were default.

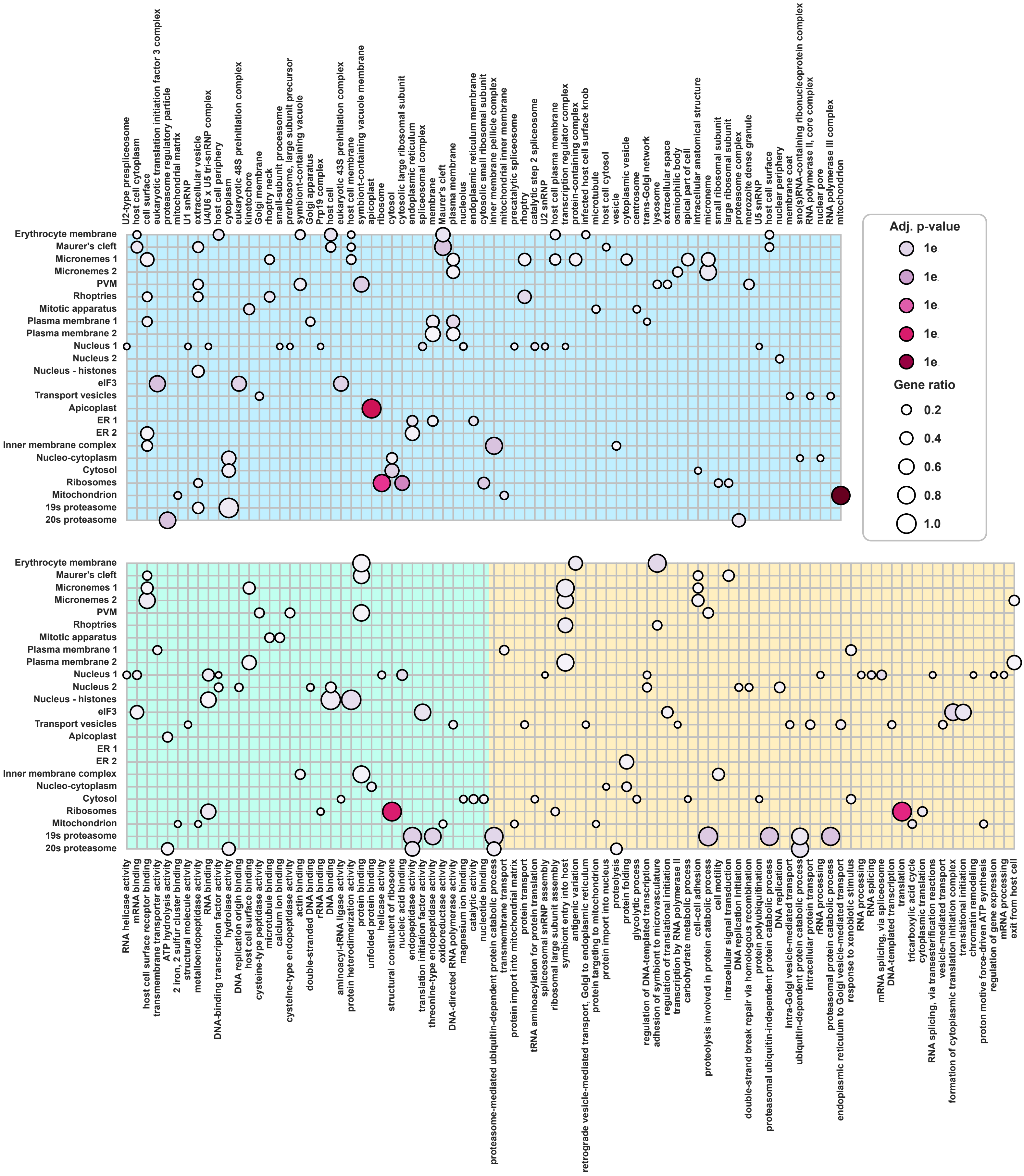

Figure S3. GO enrichment analysis of organelle proteomes

Bubble plot showing the results of Gene Ontology (GO) enrichment analysis. GO terms (x-axis) from the proteins in each compartment (y-axis) were analysed with clusterProfiler. Only significant terms (adjusted p-value ≤ 0.01) are shown, size of the dots represents the proportion of proteins showing a specific term against the total number of proteins in each compartment. GO categories are shown as follows: Cellular Component in blue, Molecular Function in green and Biological Process in yellow

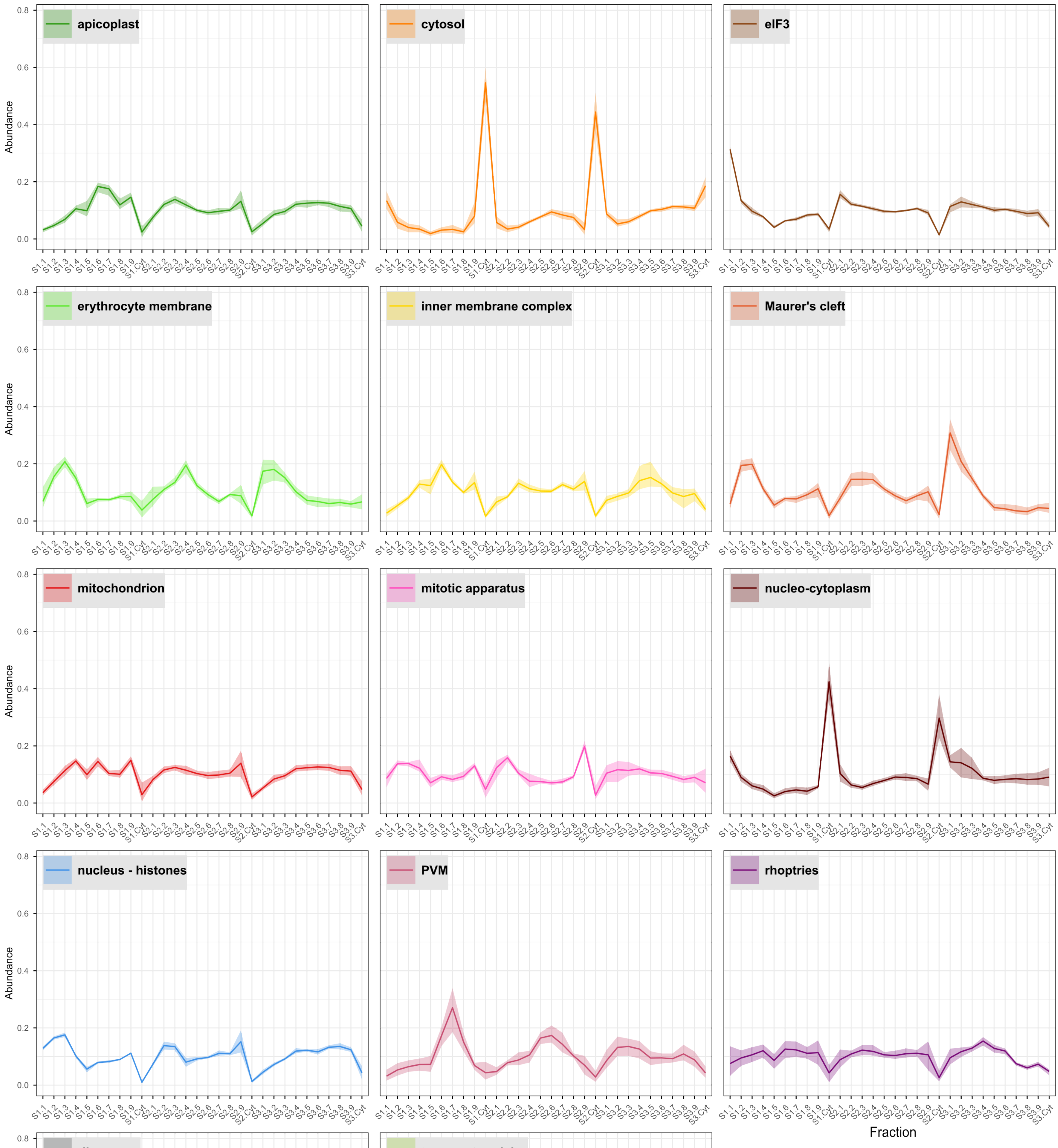

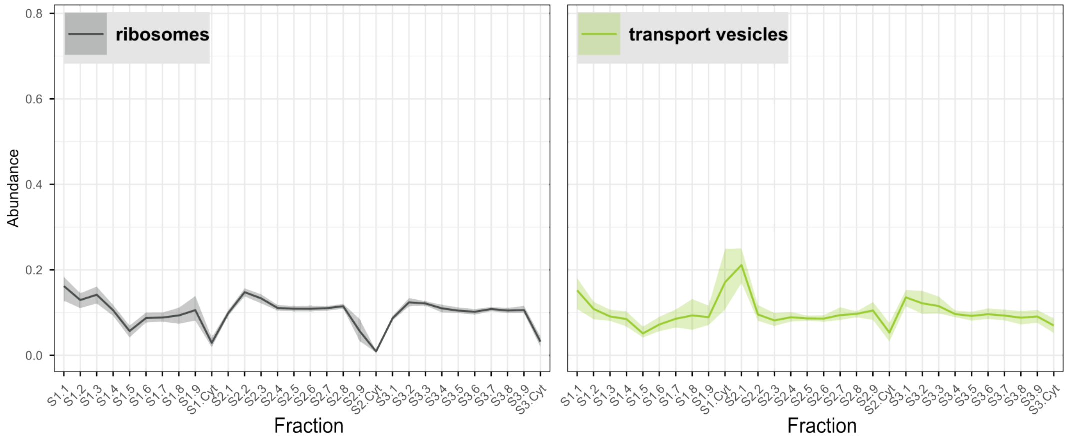

Figure S4. Abundance distribution profiles of single organelle marker proteins.
Normalised abundance profiles are shown for marker proteins across three independent hyperLOPIT experiments (S1-S2-S3), each analysed by TMT labelling. In each experiment, nine fractions from an iodixanol density gradient (S1.1–S1.9, S2.1–S2.9 and S3.1–S3.9) and a cytosolic fraction (S1.Cyt, S2.Cyt and S3.Cyt) were each labelled with an individual TMT channel. Profiles from the three experiments were concatenated to generate a combined dataset. For each organelle, the median profile is shown as a thick line with the 10th-90th percentile range shaded.

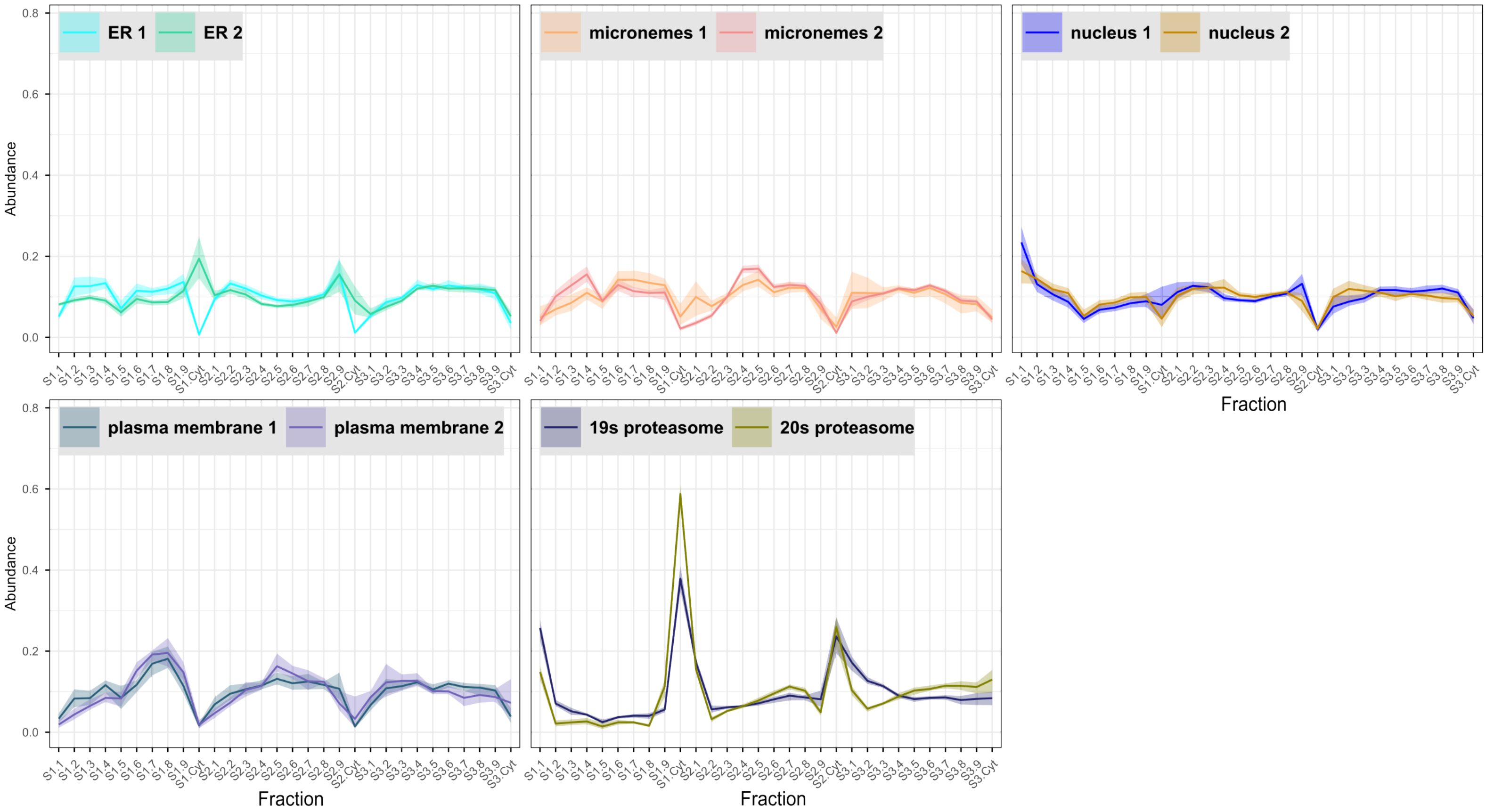

Figure S5. Abundance distribution profiles of multi-cluster organelle marker proteins. Normalised abundance profiles for marker proteins of organelles resolved as multiple clusters in the hyperLOPIT data. Data is presented as described for Figure S4. Individual subclusters are highlighted in different colours within each organelle panel. In each case, the median profile is shown as a thick line with the 10th-90th percentile range shaded.

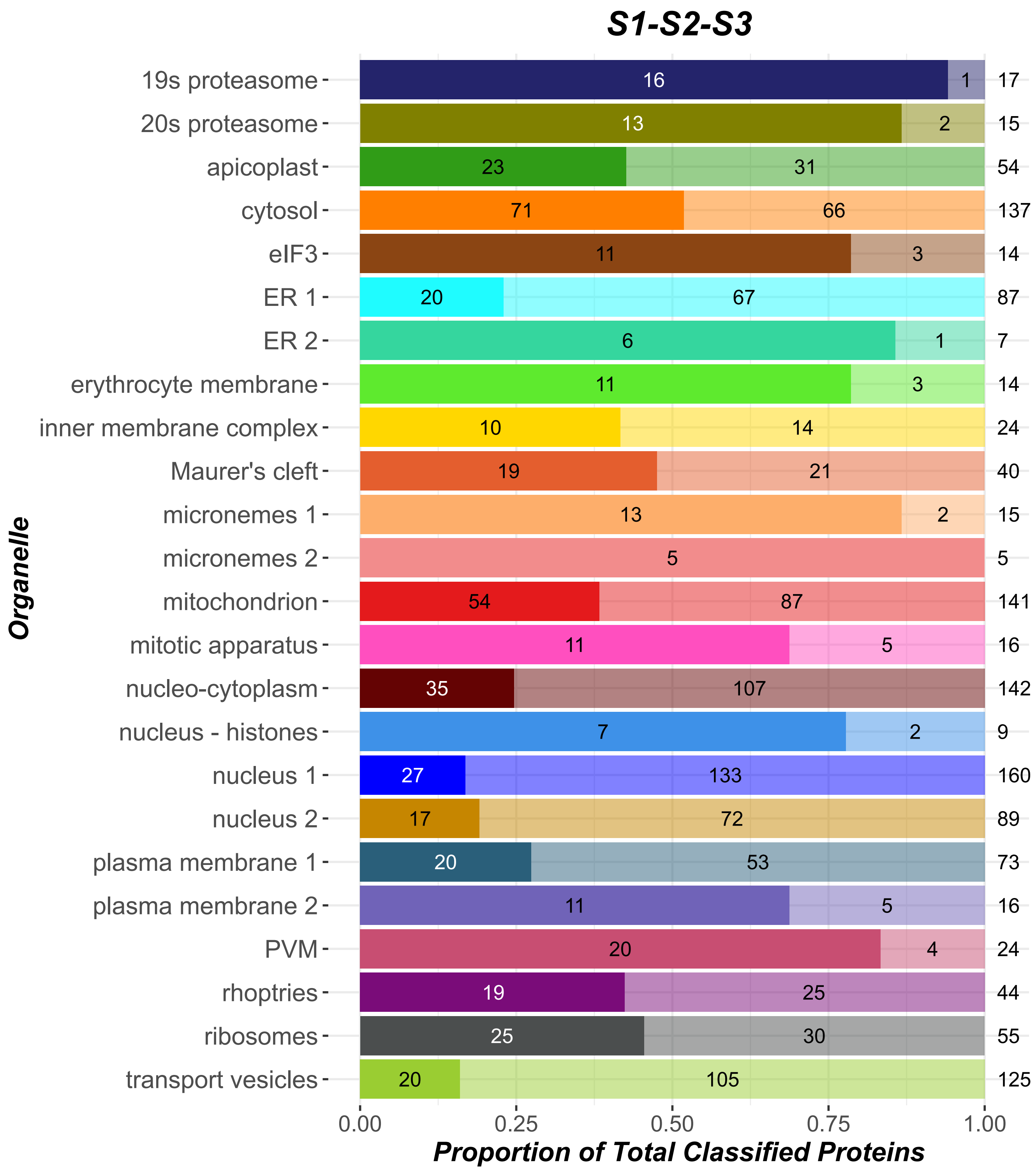

Figure S6. Results of supervised machine learning on S1-S2-S3
Numbers of proteins assigned to each compartment by Support Vector Machine (SVM) classification from the S1-S2-S3 analysis shown as bars with markers numbers to the left and predicted proteins to the right of the bars (totals at far right).

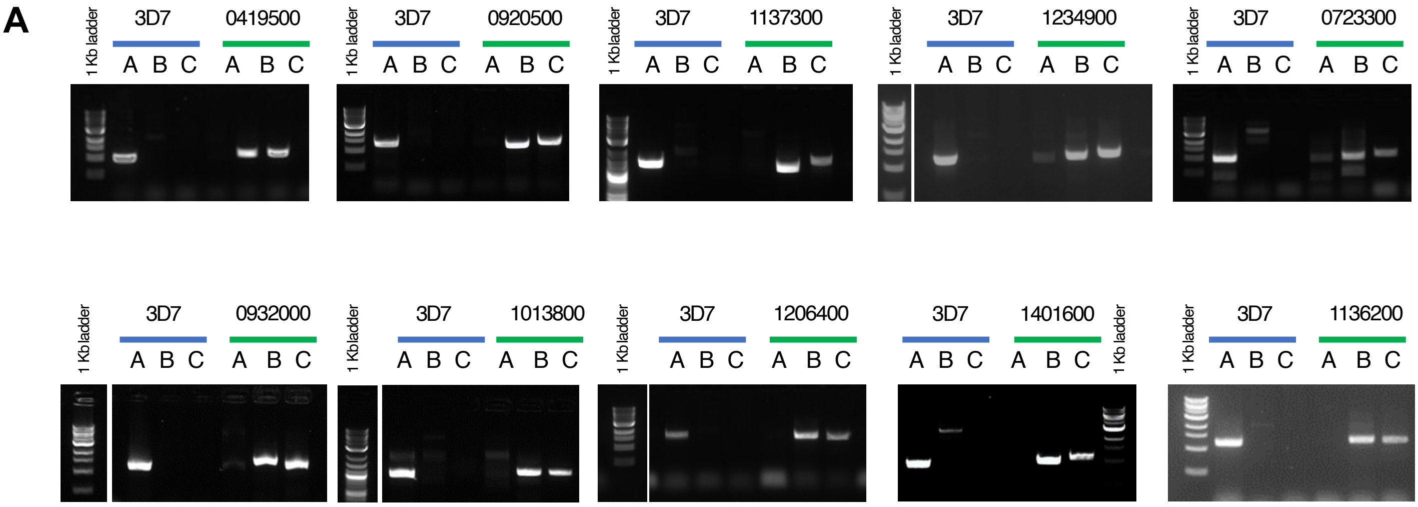

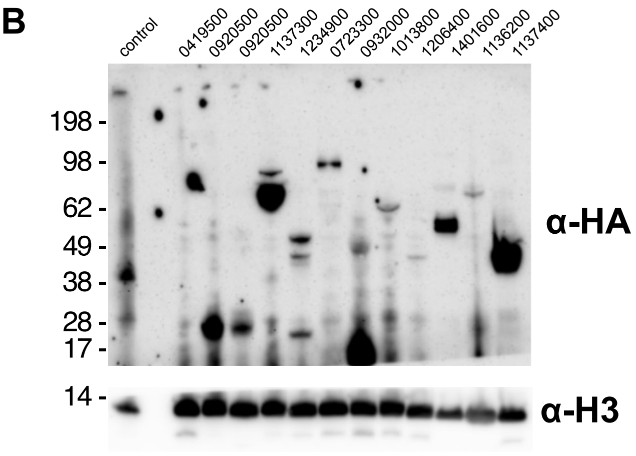

Accession	Predicted location	Predicted MW, kDa
0419500	PM	64.0
0920500	IMC	22.1
1137300	ER	81.6
1234900	Apicoplast	16.2
0723300	RBC membrane	101.1
0932000	IMC	11.0
1013800	Rhoptries	58.4
1206400	Apicoplast	41.9
1401600	Rhoptries	55.8
1136200	Rhoptries	76.6
1137400	Mitochondrion	48.6

Figure S7. Validation of C-terminal 3×HA tagging and genomic integration of candidate proteins in *Plasmodium falciparum*.
(A) Diagnostic PCRs confirming correct integration of the tagging cassette at the endogenous locus for each target gene. Lane A tests amplification of the wild-type (WT) locus, lane B tests 5′ integration, and lane C tests 3′ integration. Successful integration is indicated by amplification of both 5′ and 3′ integration products and loss of the WT band. Representative results from a single experiment are shown for each transfectant line.

Sample name	Experiment name	TMT tag name	Gradient fraction	Refractive index (˚Brix)	Peptide amount (μg)
**S1.1**	SL1	TMT127N	3 and 4 pooled	37.3	92.3
**S1.2**	SL1	TMT127C	5	30.2	68.9
**S1.3**	SL1	TMT128N	6	27.9	93.8
**S1.4**	SL1	TMT128C	7	26.7	84.2
**S1.5**	SL1	TMT129N	8	24	57.8
**S1.6**	SL1	TMT129C	9 and 10 pooled	22	58.8
**S1.7**	SL1	TMT130N	11 and 12 pooled	19.5	33.1
**S1.8**	SL1	TMT130C	13 to 17 pooled	16.2	36.5
**S1.9**	SL1	TMT131N	18 to 22 pooled	12.6	32.1
**S1.Cyt**	SL1	TMT131C	soluble	8.8	100
**S2.1**	SL2	TMT127N	3 and 4 pooled	39	100
**S2.2**	SL2	TMT127C	5 and 6 pooled	33	100.5
**S2.3**	SL2	TMT128N	7 and 8 pooled	28.8	100
**S2.4**	SL2	TMT128C	9 and 10 pooled	25.4	100
**S2.5**	SL2	TMT129N	11 and 12 pooled	22.3	92.7
**S2.6**	SL2	TMT129C	13 and 14 pooled	19.7	43.6
**S2.7**	SL2	TMT130N	15 and 16 pooled	17.3	37.4
**S2.8**	SL2	TMT130C	17 to 19 pooled	14.5	52.2
**S2.9**	SL2	TMT131C	20 to 22 pooled	11.9	47.7
**S2.Cyt**	SL2	TMT131N	soluble	8.8	100
**S3.1**	SL3	TMT127N	4 and 5 pooled	37.9	42.4
**S3.2**	SL3	TMT127C	6	34.5	35.3
**S3.3**	SL3	TMT128N	7	32.5	37.4
**S3.4**	SL3	TMT128C	8	30	48.2
**S3.5**	SL3	TMT129N	9	28.6	33.2
**S3.6**	SL3	TMT129C	10 to 12 pooled	25.5	66
**S3.7**	SL3	TMT130N	13 and 14 pooled	22.1	52.5
**S3.8**	SL3	TMT130C	15 to 18 pooled	18.6	45.1
**S3.9**	SL3	TMT131N	19 to 22 pooled	13.9	20.5
**S3.Cyt**	SL3	TMT131C	soluble	8.8	52.1

Supplementary Table 1: TMT channel assignments for hyperLOPIT experiments
Description: Summary of TMT 11-plex channel assignments for gradient fractions across three independent hyperLOPIT experiments (S1, S2, S3), indicating which density gradient fractions and cytosolic samples were labelled with each TMT channel.

Organism	Strain	Genome size (Mbp)	CDS	BUSCO (%)
Babesia bovis	T2Bo	8.22	3974	99.4
Cardiosporidium cionae	ESH_2018	57.03	4566	80.7
Cryptosporidium parvum	Iowa II	9.1	3941	96.7
Cyclospora cayetanensis	HEN01	44.03	7153	74.8
Eimeria tenella	Houghton	51.85	7268	99
Haemoproteus tartakovskyi	SISKIN1	23.2	4860	67.2
Hepatocystis piliocolobus	2019	19.98	5341	92.9
Neospora caninum	Liverpool 2019	61.49	7364	98.3
Nephromyces	MM_2019	59.89	10628	80.5
Plasmodium berghei	ANKA	18.78	4958	99.8
Plasmodium chabaudi	chabaudi	18.97	5199	100
Plasmodium falciparum	3D7	23.33	5318	99.6
Plasmodium gaboni	SY75	20.38	5198	95.4
Plasmodium gallinaceum	8A	25.03	5286	99.6
Plasmodium knowlesi	H	24.39	5328	100
Plasmodium reichenowi	CDC	24.05	5638	99.6
Plasmodium vivax	P01	29.04	6523	99.8
Plasmodium yoelii	17X	23.08	6040	99.8
Sarcocystis neurona	SN3	124.41	6965	86.1
Theileria annulata	Ankara	8.35	3795	97.3
Toxoplasma gondii	ME49	65.66	8322	99.6

Supplementary Table 2: Species used in orthology analysis
Description: List of 21 organisms used for orthology and evolutionary analyses, including species names, taxonomic classifications, and data sources. Except in the cases of *Cardiosporidium* (accession number GCA_015476325.1) and *Nephromyces* (accession number GCA_015657535.1), all sequences were collected from vEuPathDB release 68. Completeness of the datasets was performed using BUSCO v5.8.3 against the apicomplexa_ODB12 dataset.
