# Supplementary material for: The spatial proteome of the Plasmodium falciparum schizont illuminates the composition and evolutionary trajectories of its organelles
Source: Nat Commun. 2026 May 30;17:6192. doi: 10.1038/s41467-026-73664-2 (PMC13369866; doi:10.1038/s41467-026-73664-2)
Supplement: Supplementary file 2 — Description of Additional Supplementary File [file 41467_2026_73664_MOESM2_ESM.pdf]

Description of additional supplementary files

Supplementary Data 1: Complete hyperLOPIT dataset and protein classifications Complete dataset of 3,000 *P. falciparum* proteins detected across three hyperLOPIT experiments (S1, S2, S3). Includes protein identifiers, compartment assignments and SVM scores, HDBSCAN cluster assignments, MalariaGEN variant metrics, evolutionary analyses (pN/pS, dN/dS, evolutionary origin), disorder predictions, and Gene Ontology annotations.

Supplementary Data 2: Oligonucleotide primers, antibodies, and reagents
List of oligonucleotide primers used for cloning and diagnostic PCR, antibodies used for immunofluorescence assays and Western blotting and their sources and working concentrations, and reagents used in this study.

Supplementary Data 3: MalariaGEN variant analysis and evolutionary metrics
Description: Evolutionary and genomic variant analysis for all *P. falciparum* genes in the MalariaGEN Pf v7.0 dataset (20,864 isolates), used for comparative analyses in Figures 5 and 6. Includes dN/dS ratios for orthologs within the *Laverania* group and broader *Plasmodium* genus (average, minimum, maximum values with ortholog counts), adjusted pN/pS ratios, and variant fractions.
