# Supplementary material for: The spatial proteome of the Plasmodium falciparum schizont illuminates the composition and evolutionary trajectories of its organelles
Source: Nat Commun. 2026 May 30;17:6192. doi: 10.1038/s41467-026-73664-2 (PMC13369866; doi:10.1038/s41467-026-73664-2)
Supplement: Supplementary file 6 — Reporting summary [file 41467_2026_73664_MOESM6_ESM.pdf]

nature portfolio

Corresponding author(s): Ross F Waller, Scott A Chisholm

Last updated by author(s): Apr 24, 2026

Reporting Summary

Nature Portfolio wishes to improve the reproducibility of the work that we publish. This form provides structure for consistency and transparency in reporting. For further information on Nature Portfolio policies, see our Editorial Policies and the Editorial Policy Checklist.

Statistics

For all statistical analyses, confirm that the following items are present in the figure legend, table legend, main text, or Methods section.

n/a	Confirmed	
☐	☒	The exact sample size (*n*) for each experimental group/condition, given as a discrete number and unit of measurement
☐	☒	A statement on whether measurements were taken from distinct samples or whether the same sample was measured repeatedly
☐	☒	The statistical test(s) used AND whether they are one- or two-sided<br>*Only common tests should be described solely by name; describe more complex techniques in the Methods section.*
☒	☐	A description of all covariates tested
☐	☒	A description of any assumptions or corrections, such as tests of normality and adjustment for multiple comparisons
☐	☒	A full description of the statistical parameters including central tendency (e.g. means) or other basic estimates (e.g. regression coefficient) AND variation (e.g. standard deviation) or associated estimates of uncertainty (e.g. confidence intervals)
☐	☒	For null hypothesis testing, the test statistic (e.g. *F*, *t*, *r*) with confidence intervals, effect sizes, degrees of freedom and *P* value noted<br>*Give P values as exact values whenever suitable.*
☒	☐	For Bayesian analysis, information on the choice of priors and Markov chain Monte Carlo settings
☒	☐	For hierarchical and complex designs, identification of the appropriate level for tests and full reporting of outcomes
☒	☐	Estimates of effect sizes (e.g. Cohen's *d*, Pearson's *r*), indicating how they were calculated

Our web collection on statistics for biologists contains articles on many of the points above.

Software and code

Policy information about availability of computer code

Data collection	Data was collected using Proteome Discoverer (Thermo Scientific, version 3.1). No further data collection software was used in this study
Data analysis	R v4.5.0 (R Core Team, 2024); MSnbase v2.34.0 (Gatto and Lilley, 2012); pRoloc v1.48.0 (Breckels et al., 2016); tidyverse v2.0.0; Proteome Discoverer v3.1 (Thermo Fisher Scientific); XCalibur v4.2.47 (Thermo Fisher Scientific); bcftools v1.11 (Danecek et al., 2021); SnpEff v5.21f (Cingolani, 2022); clusterProfiler v4.16.0 (Xu et al., 2024); OrthoFinder v2.5.5 (Emms and Kelly, 2019); mcl v14.137; mafft v7.475 (Nakamura et al., 2018); iqtree2 (Minh et al., 2020); PAML v4.10.6 (Yang, 2007); iupred3 (Erdos et al., 2021); MobiDB-lite v3.9.0 (Necci et al., 2017); DSSP v2.3.0 (Kabsch and Sander, 1983); Fiji/ImageJ (Schindelin et al., 2012); Leica LAS X; ete3 toolkit (Huerta-Cepas et al., 2016); pal2nal (Suyama et al., 2006); OrthoPrep v0.0.1 (https://github.com/vflorelo/orthoprep); diamond (Buchfink et al., 2015).

For manuscripts utilizing custom algorithms or software that are central to the research but not yet described in published literature, software must be made available to editors and reviewers. We strongly encourage code deposition in a community repository (e.g. GitHub). See the Nature Portfolio guidelines for submitting code & software for further information.

Data

Policy information about availability of data

All manuscripts must include a data availability statement. This statement should provide the following information, where applicable:

- Accession codes, unique identifiers, or web links for publicly available datasets
- A description of any restrictions on data availability
- For clinical datasets or third party data, please ensure that the statement adheres to our policy

The mass spectrometry proteomics data have been deposited to the ProteomeXchange Consortium via the PRIDE partner repository with the dataset identifier PXD070842 and 10.6019/PXD070842. The data are integrated into PlasmoDB.org. An interactive interface to the annotated spatial proteome data is available via a web-based R Shiny application at https://proteome.shinyapps.io/plasmolopitsz/ (S1-S2) and https://proteome.shinyapps.io/plasmolopitszmz (S1-S2-S3).

Research involving human participants, their data, or biological material

Policy information about studies with human participants or human data. See also policy information about sex, gender (identity/presentation), and sexual orientation and race, ethnicity and racism.

Reporting on sex and gender	Not relevant for this study
Reporting on race, ethnicity, or other socially relevant groupings	Not relevant for this study
Population characteristics	Not relevant for this study
Recruitment	No human participants were involved in this study
Ethics oversight	NHS Cambridge South Research Ethics Committee (20/EE/0100) and the University of Cambridge Human Biology Research Ethics Committee (HBREC.2019.40).

Note that full information on the approval of the study protocol must also be provided in the manuscript.

Field-specific reporting

Please select the one below that is the best fit for your research. If you are not sure, read the appropriate sections before making your selection.

☒ Life sciences ☐ Behavioural & social sciences ☐ Ecological, evolutionary & environmental sciences

For a reference copy of the document with all sections, see nature.com/documents/nr-reporting-summary-flat.pdf

Life sciences study design

All studies must disclose on these points even when the disclosure is negative.

Sample size	Sample size was determined by the number of gradient fractions analyzed (n=30 across three 10plex experiments for machine learning) and by the number of proteins assigned to each organelle class for organelle-specific analyses
Data exclusions	TMT126 (densest gradient fraction) was excluded from analysis of all three hyperLOPIT experiments as its inclusion decreased overall resolution, likely due to peptide co-fractionation with ribosomal subunits
Replication	The hyperLOPIT experimental design relies on the consistency of protein abundance distribution profiles across fractionation gradients from samples generation methods to assign a likelihood of colocation in the cell and, therefore, sample replicates are not relevant or used.
Randomization	Samples were processed systematically rather than randomized as hyperLOPIT is a descriptive technique for mapping protein subcellular localization without treatment group comparisons
Blinding	Blinding was not applicable as hyperLOPIT data acquisition relies on automated mass spectrometry measurements that are independent of experimenter knowledge of sample identity

Reporting for specific materials, systems and methods

We require information from authors about some types of materials, experimental systems and methods used in many studies. Here, indicate whether each material, system or method listed is relevant to your study. If you are not sure if a list item applies to your research, read the appropriate section before selecting a response.

Materials & experimental systems

n/a	Involved in the study
☐	☒ Antibodies
☒	☐ Eukaryotic cell lines
☒	☐ Palaeontology and archaeology
☒	☐ Animals and other organisms
☒	☐ Clinical data
☒	☐ Dual use research of concern
☒	☐ Plants

Methods

n/a	Involved in the study
☒	☐ ChIP-seq
☒	☐ Flow cytometry
☒	☐ MRI-based neuroimaging

Antibodies

Antibodies used

Antibodies used in this study, including their sources, are listed in Supplementary Information 2.

Validation

Commericially supplied antibodies were validated for expected molecular weight by western blot. Previously published antibodies (KAHRP, RAP1, MTIP, CPN60) were not validated as their activity and conditions had been already established. This is all summarised in Supplementary Information 2.

Plants

Seed stocks

Not relevant for this study

Novel plant genotypes

Not relevant for this study

Authentication

Not relevant for this study
