# Supplementary material for: The spatial proteome of the Plasmodium falciparum schizont illuminates the composition and evolutionary trajectories of its organelles
Source: Nat Commun. 2026 May 30;17:6192. doi: 10.1038/s41467-026-73664-2 (PMC13369866; doi:10.1038/s41467-026-73664-2)
Supplement: Supplementary file 7 — Transparent Peer Review file [file 41467_2026_73664_MOESM7_ESM.pdf]

nature portfolio

# Peer Review File

# The spatial proteome of the *Plasmodium falciparum* schizont illuminates the composition and evolutionary trajectories of its organelles

Corresponding Author: Professor Ross Waller

**This file contains all reviewer reports in order by version, followed by all author rebuttals in order by version.**

Version 0:

Reviewer comments:

Reviewer #1

(Remarks to the Author)
This study by Chisholm et al. provides key insights into the P. falciparum proteome and offers a valuable resource for the Plasmodium research community. The study offers the first high-resolution spatial proteome map of the Plasmodium falciparum schizont stage, identifying 24 distinct subcellular compartments and classifying 1,646 proteins into these compartments. This map provides critical insights into the localization of both conserved and novel proteins, providing a framework for further functional investigations. To validate the location assignments, nine previously uncharacterized proteins were tagged and localized by IFA, matching the hyperLOPIT assignments. Additional evolutionary insights are provided. It is worth noting that the Waller group has previously used hyperLOPIT to map the spatial proteomes of T. gondii and Cryptosporidium, which lends a measure of confidence to the method and analyses when there is so little else to compare the results with. Inherent concerns lie within the confidence of the location assignments of the 1646 classified proteins as there is little to no discussion of the certainty of the classifications or how they should utilized by the community. Following up on one or more newly identified protein(s) with functional characterization would boost the impact of the manuscript.

Comments

Describe the inclusion/exclusion/statistical criteria for the 1646 proteins that received a compartment classification. Were specific numerical thresholds used?

Table S2 is difficult to interpret. For example, row 7: PF3D7_0103100 nucleoside transporter 4, S1-S2 final location2 (plasma membrane 1), S1-S2-S3 final location (plasma membrane 1), hdb.cluster (S1-S2) (0), hdb.probability (S1-S2) (0), hdb.cluster (S1-S2-S3) (0), hdb.probability (S1-S2-S3) (0). How was this protein assigned plasma membrane 1 if the hdb cluster is 0 and hdb probability is 0? Readers should be able to understand how these proteins were classified using the information in the table. Perhaps there should be a probability value next to each final localization columns? It would also be helpful to highlight the 1646 proteins classified by machine learning yellow, and markers highlighted green. Or organize by compartment.

Discuss the likelihood/frequency of false positive and false negative protein assignments.

It appears the compiled list of marker proteins were selected if they fit the data (i.e. clustered with other known markers of that compartment). “Overall, we compiled a list of 542 marker proteins which indicated clear segmentation of different subcellular compartments with obvious identities, including resolution of compartments within the host erythrocyte such as the PVM and Maurer’s clefts”. While this approach seems appropriate for marking clusters by majority, it makes it difficult to assess the real distribution of bona fide markers that fell outside of its matched cluster or into other clusters.

Functional insights are limited to the localization of nine novel proteins by IFA. Choose one of these proteins to characterize functionally (low fitness score). For instance, cKD/growth.

As mentioned in the discussion, a spatial proteome of Plasmodium falciparum was previously published (Siau et al. 2023

Cell Rep). Where direct comparisons are possible (e.g. pvm), discuss whether there is any consistency between the two studies for protein assignments.

Fig S4 is not legible at current size.

Discuss how these results compare with hyperLOPIT assignments published for T. gondii and Cryptosporidium in terms of methodologies and coverage.

Reviewer #2

(Remarks to the Author)
Overall Review:
The manuscript presents a comprehensive compartment proteomics analysis of the Plasmodium falciparum schizont stage, providing significant insights into the subcellular localization of proteins and their roles in parasite biology and evolution. The application of hyperplexed Localisation of Organelle Proteins by Isotopic Tagging (hyperLOPIT) is robust, and the results contribute valuable knowledge to the field. However, the following adjustments are requested to improve clarity and address some specific methodological concerns.

1) TMT-11plex Labeling of Samples: It is noted that each TMT experiment appears to be missing a channel that could be used for labeling a universal pooled sample. Utilizing one channel for a pooled internal standard across all experiments would greatly facilitate the normalization and quantification of data across the different experiments, thereby increasing the robustness and comparability of the results. Clarify why one channel in the TMT-11plex was not utilized for a universal pooled sample.

2) Exclusion of the S3 Sample in Figure 2 D&E: Figure 2 D&E presents results excluding the S3 sample. The exclusion of this sample might introduce biases or reduce the comprehensiveness of the data interpretation. Please provide a rationale for excluding the S3 sample in the context of those figures, perhaps adding a brief discussion of how this exclusion impacts the results and their reliability.

3) Single Cell Proteomics: Single-cell proteomics on red blood cells (RBCs) might be crucial for this type of study because of the unique and significant biological insights it can provide. Infected RBCs can exhibit significant heterogeneity in terms of parasite load, stage of schizont development, and expression levels of both host and parasite proteins. Single-cell proteomics of an RBC could allow for the detailed analysis of these variations, providing a granular view of how the parasite interacts with the host cell at different stages of infection. Including the authors' perspective on this front in the discussion would be beneficial, as single-cell proteomics is very feasible nowadays.

4) Is this really a spatial analysis? The density gradient doesn't mean anything is being spatially visualized within the cells. Isolated compartments through this technique only gives fractionation of cells to be analyzed by TMT proteomics. Because of this, the title of the paper is slightly misleading as this is not truly a spatial technique.

Additional Minor Comments:
1.Discussion: Adding a few more references to comparable studies using isolated single-cell proteomics might strengthen the discussion on methodological parallels and distinctions. This will contextualize your findings within the broader scope of recent advances in the field.

Reviewer #3

(Remarks to the Author)
I co-reviewed this manuscript with one of the reviewers who provided the listed reports. This is part of the Nature Communications initiative to facilitate training in peer review and to provide appropriate recognition for Early Career Researchers who co-review manuscripts.

Version 1:

Reviewer comments:

Reviewer #1

(Remarks to the Author)
All concerns raised in initial peer review have been carefully and adequately addressed in the revised manuscript or by clarification/justification in the author response (rebuttal). No additional concerns were noted in the revised manuscript.

Reviewer #2

(Remarks to the Author)
I co-reviewed this manuscript with one of the reviewers who provided the listed reports. This is part of the Nature Communications initiative to facilitate training in peer review and to provide appropriate recognition for Early Career

Researchers who co-review manuscripts.

Reviewer #3

(Remarks to the Author)
I co-reviewed this manuscript with one of the reviewers who provided the listed reports. This is part of the Nature Communications initiative to facilitate training in peer review and to provide appropriate recognition for Early Career Researchers who co-review manuscripts.

**Open Access** This Peer Review File is licensed under a Creative Commons Attribution 4.0 International License, which permits use, sharing, adaptation, distribution and reproduction in any medium or format, as long as you give appropriate credit to the original author(s) and the source, provide a link to the Creative Commons license, and indicate if changes were made.
In cases where reviewers are anonymous, credit should be given to 'Anonymous Referee' and the source.
The images or other third party material in this Peer Review File are included in the article’s Creative Commons license, unless indicated otherwise in a credit line to the material. If material is not included in the article’s Creative Commons license and your intended use is not permitted by statutory regulation or exceeds the permitted use, you will need to obtain permission directly from the copyright holder.
To view a copy of this license, visit https://creativecommons.org/licenses/by/4.0/

**REVIEWER COMMENTS**

In addition to our corrections and responses to all reviewer comments below, we also note that we detected a minor error in labelling of the proteasome subcompartments in our report where the 19S and 20S components had been reciprocally mislabelled. We note that this was a labelling error only and the underlying data, protein assignments, and biological interpretations remain unchanged and accurate. This labelling has been corrected throughout, and we apologise for this minor oversight.

**Reviewer #1** (Remarks to the Author):

This study by Chisholm et al. provides key insights into the P. falciparum proteome and offers a valuable resource for the Plasmodium research community. The study offers the first high-resolution spatial proteome map of the Plasmodium falciparum schizont stage, identifying 24 distinct subcellular compartments and classifying 1,646 proteins into these compartments. This map provides critical insights into the localization of both conserved and novel proteins, providing a framework for further functional investigations. To validate the location assignments, nine previously uncharacterized proteins were tagged and localized by IFA, matching the hyperLOPIT assignments. Additional evolutionary insights are provided. It is worth noting that the Waller group has previously used hyperLOPIT to map the spatial proteomes of T. gondii and Cryptosporidium, which lends a measure of confidence to the method and analyses when there is so little else to compare the results with. Inherent concerns lie within the confidence of the location assignments of the 1646 classified proteins as there is little to no discussion of the certainty of the classifications or how they should utilized by the community. Following up on one or more newly identified protein(s) with functional characterization would boost the impact of the manuscript.

Comment 1.1
Describe the inclusion/exclusion/statistical criteria for the 1646 proteins that received a compartment classification. Were specific numerical thresholds used?

Author response: We thank the reviewer for raising this point, and have addressed this issue with modification to both the Results text and Methods. Our classification criteria are described in detail in the Methods section (lines 889-899). Following SVM modelling on the 542 curated marker proteins, we generated compartment allocations and SVM scores for all proteins. We initially applied a threshold based on the median SVM score per protein as our baseline classification criterion, as is common practice. To improve specificity and reduce false positives, we manually refined thresholds for ten compartments where initial assignments appeared overly permissive. These refinements were guided by SVM score distributions and existing evidence in the literature, ApiLoc, and PlasmoDB, and sought to minimise false assignments. The following thresholds were adjusted from baseline: 20S proteasome (0.35 → 0.60), micronemes 1 (0.24 → 0.28), ER 1 (0.37 → 0.43), Maurer's clefts (0.33 → 0.34), plasma membrane 1 (0.38 → 0.49), ER 2 (0.22 → 0.30), transport vesicles (0.17 → 0.35), nucleus 1 (0.22 → 0.50), and nucleus 2 (0.22 → 0.42). This approach balanced SVM classification with biological knowledge to ensure robust protein localisations whilst minimising over-classification. The details for these adjustments is given in the Methods (Lines: 892-899 ).

Comment 1.2
Table S2 is difficult to interpret. For example, row 7: PF3D7_0103100 nucleoside transporter 4, S1-S2 final location2 (plasma membrane 1), S1-S2-S3 final location (plasma membrane 1), hdb.cluster (S1-S2) (0), hdb.probability (S1-S2) (0), hdb.cluster (S1-S2-S3) (0), hdb.probability (S1-S2-S3) (0). How was this protein assigned plasma membrane 1 if the hdb cluster is 0 and hdb probability is 0? Readers should be able to understand how these

proteins were classified using the information in the table. Perhaps there should be a probability value next to each final localization columns? It would also be helpful to highlight the 1646 proteins classified by machine learning yellow, and markers highlighted green. Or organize by compartment.

Author response: We apologise that the table lacked some key information for interpreting protein classifications, and some superfluous information. The HDBSCAN clustering columns (hdb.cluster and hdb.probability) represent an unsupervised clustering analysis used to identify potential compartments in the data (Figure S2), but final protein assignments were made using Support Vector Machine (SVM) analysis with curated marker proteins as training data, as described in the Methods. The absence of SVM scores in the original table was an accidental omission and obscured this distinction.

We have revised Table S2 as follows: (1) added SVM scores for each protein's assigned compartment in both the S1-S2 and S1-S2-S3 analyses, allowing readers to assess classification confidence; (2) removed the HDBSCAN probability columns, which were not used for final classifications; and (3) retained HDBSCAN cluster assignments for additional context on data structure.

The 1646 proteins that are classified by SVM are all indicated in Table S2, including their assigned locations, in column 5 ('Final location (S1S2)'), as are the assignments made from the combined S1-S2-S3 analysis (column 9). Marker proteins are indicated in columns 4 and 8. Readers will be free to annotate these proteins (e.g. with colour coding) as suits them best in this editable data resource. But we think it is better to leave the data in this table in its unannotated from to allow readers to further draw their own interpretations such as for proteins close to the high-confidence values that we have used.

Comment 1.3
Discuss the likelihood/frequency of false positive and false negative protein assignments.

Author response: We thank the reviewer for this important consideration. As with any machine learning-based classification, our assignments are subject to both false positive and false negative errors.

**False negatives:** Our classification strategy prioritised specificity over sensitivity by applying conservative SVM score thresholds (detailed in our response to Comment 1.1). We therefore expect false negatives to be more frequent than false positives and many proteins that genuinely localise to specific compartments were likely left unassigned due to borderline scores falling below our thresholds. We estimate that several hundred additional proteins could be confidently assigned with more permissive thresholds or additional marker proteins. Readers can explore potential false negatives in Table S2 by examining SVM scores, or via our interactive web applications (https://proteome.shinyapps.io/plasmolopitsz/ and https://proteome.shinyapps.io/plasmolopitszmz/)

**False positives:** While our conservative thresholds and manual refinement were designed to minimise false positives, some misclassifications are inevitable. Quantifying the false positive rate is challenging without systematic experimental validation. Our immunofluorescence validation of 9 previously uncharacterised proteins (Figure 3) showed 100% concordance with hyperLOPIT predictions, though this sample is too small to extrapolate a genome-wide false positive rate. We expect that false positives likely represent a small fraction of the 1646 classified proteins, but readers should interpret individual assignments alongside other available evidence.

Overall, our approach favours high-confidence assignments at the cost of leaving many proteins unclassified. We have included additional text (Lines 233-238, 489-501, 891-899) to

make these objectives clearer and to discuss the possible causes of false negatives and positives.

We encourage users to explore the complete dataset to identify additional candidates for their compartments of interest.

Comment 1.4
It appears the compiled list of marker proteins were selected if they fit the data (i.e. clustered with other known markers of that compartment). “Overall, we compiled a list of 542 marker proteins which indicated clear segmentation of different subcellular compartments with obvious identities, including resolution of compartments within the host erythrocyte such as the PVM and Maurer’s clefts”. While this approach seems appropriate for marking clusters by majority, it makes it difficult to assess the real distribution of bona fide markers that fell outside of its matched cluster or into other clusters.

Author response: This reviewer has raised an important methodological point which we now discuss. Marker curation strategies are informed by abundance distribution profiles in the hyperLOPIT data by design. This was necessary to ensure robust machine learning classification. The purpose of marker proteins in supervised learning is to provide reliable training data that defines compartment boundaries in high-dimensional space. We therefore required both strong literature support (from multiple sources including literature, ApiLoc, and PlasmoDB) and consistency of abundance distribution profiles with other known members of each compartment. Proteins with established localisations but profiles inconsistent with the majority of compartment members were excluded from the marker set to optimise classifier training, though these proteins remained in the dataset for SVM prediction.

This exclusion does not indicate incorrect literature annotations. Rather, inconsistent profiles may reflect biological and/or technical complexity such as dual localisation, transient associations, stage-specific behaviour not captured under our experimental conditions, or variability in the measurability of the protein peptides by mass spectometry. Importantly, excluded proteins were not excluded from classification. They remain in the dataset and were subjected to SVM prediction alongside all other proteins. Some received classifications consistent with existing knowledge, whilst others were left unassigned or assigned to different compartments, potentially revealing genuine biological insights or highlighting technical limitations for certain protein classes.

We have clarified this strategy in the Methods (lines 875-880) to make explicit that marker selection was optimised for classifier performance whilst maintaining biological validity.

Comment 1.5
Functional insights are limited to the localization of nine novel proteins by IFA. Choose one of these proteins to characterize functionally (low fitness score). For instance, cKD/growth.

Author response: We have not sought to functionally characterise proteins as part of this study because its primary goal is to provide a cell-wide measure of the location of proteins, including the many uncharacterised novel proteins, within the cell. The location studies that we have performed were not intended to constitute functional studies, rather to independently test the accuracy of the hyperLOPIT location predictions. Gene knockout data is already available at a genome wide level from other published studies and this information can readily be overlayed on our spatial maps. Indeed, we’ve analysed these data already with respect to the distribution of relative phenotypes of protein disruptions (Figure 5).

The scale of a meaningful functional characterisation of one or multiple proteins is beyond what is feasible in this report. In fact, we have used our hyperLOPIT data already in a

companion study (Paoletta et al. bioRxiv, 2025, https://doi.org/10.64898/2025.12.21.695507) that identifies the role of two of the exported proteins in the cytoadherence of infected erythrocytes in the microvasculature of their hosts. This work clearly demonstrates the utility of this resource for guiding targeted functional studies, and the scale of what such a task represents (for instance, seven composite figures of data in this example).

We have made the complete dataset freely available via Table S2, interactive web applications (https://proteome.shinyapps.io/plasmolopitsz/ and https://proteome.shinyapps.io/plasmolopitszmz/) and submitted it to the widely used PlasmoDB.org, specifically to enable researchers to identify candidate proteins for functional studies in their compartments of interest. We anticipate this resource will facilitate many such investigations by the broader *Plasmodium* research community.

Comment 1.6

As mentioned in the discussion, a spatial proteome of Plasmodium falciparum was previously published (Siau et al. 2023 Cell Rep). Where direct comparisons are possible (e.g. pvm), discuss whether there is any consistency between the two studies for protein assignments.

Author response: The Siau et al. (2023) study "**Comparative spatial proteomics of Plasmodium-infected erythrocytes**" focused specifically on protein locations in the host erythrocyte compartments, rather than the internal locations with *Plasmodium* cells, so is substantially different from our study. In their study they used sequential detergent extraction coupled with mass spectrometry to fractionate proteins across five *Plasmodium* species, providing a valuable complementary dataset. We believe that direct tabular comparison of protein assignments between studies would be of limited utility, as the two methods have fundamentally different aims. Nevertheless, to address the reviewer's question we have mapped the most relevant of the Siau et al. predictions onto our hyperLOPIT t-SNE plots for two compartments (Figure attached), using the interactive web tool that is also openly available to readers.

In the Siau study the 'ghost' protein fraction, which should enrich for the host cell membrane and Maurer's clefts, indeed overlap with these two clusters in our maps. However, the Siau et al. Ghost predictions include several proteins with established alternative localisations. Examples include PHIL1 (PF3D7_0109000), an established inner membrane complex protein (PMID: 29138437); HSP101 (PF3D7_1116800), a known PV protein (PMID: 19536257); bromodomain protein 5 (PF3D7_1234100), a known nuclear protein (PMID: 31728527); and GET4 (PF3D7_1438600), known to be internal to the parasite (PMID: 34780541). Additionally, 10 putative ribosomal proteins and 4 components of eIF3 machinery were assigned to Ghost, suggesting substantial contamination from the abundant cytosolic translation machinery and thus internal parasite compartments. This pattern of enrichment of ribosomal proteins in unexpected compartments appears throughout the Siau et al. dataset and likely reflects a fundamental limitation of sequential extraction methods when dealing with highly abundant protein complexes.

Similarly, while many of the PVM predictions from Siau et al. overlap with our PVM cluster, the Siau data include two rifins (PF3D7_1100300 and PF3D7_0401600) known to localise to the host erythrocyte surface, PiT (PF3D7_1340900), a plasma membrane-localised transporter (PMID: 17006451), a putative proteasome regulatory subunit (PF3D7_1414000), a putative DNA repair protein (PF3D7_0605800), and kinetochore protein 6 (PF3D7_0420600), none of which have obvious biological rationale for PVM localisation.

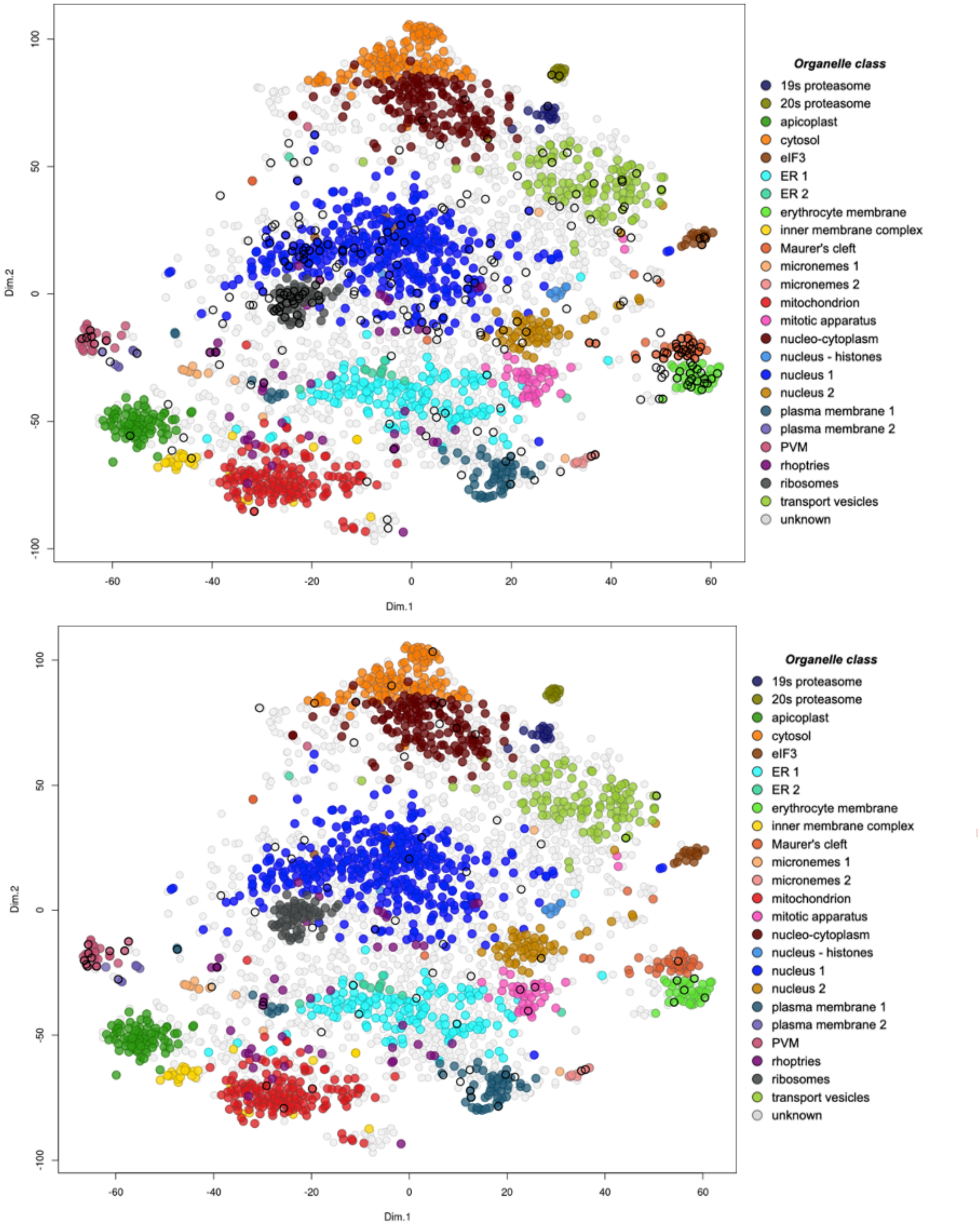


**Figure 1. Siau et al. Ghost  and PVM predictions mapped onto t-SNE plot of our hyperLOPIT data**. Siau et al. (2023) predictions for the ghost (above) and PVM (below) are indicated by black circles overlaid on the S1-S2 t-SNE plot. Proteins are coloured according to our compartment assignments as shown in the legend.

Our dataset also assigns compartmental locations to several well-characterised proteins absent or misassigned from corresponding Siau et al. predictions. For the Ghost/host cell compartment, Siau et al. failed to identify well-established exported proteins including

KAHRP (PF3D7_0202000), REX2 (PF3D7_0936000), EH2 (PF3D7_1401300), and KAHsp40 (PF3D7_0201800, PMID: 22970262), as well as many PfEMP1 and rifin proteins (though antigenic variation may explain some differences). For PVM, they missed well-established translocon components TRX2, HSP101, and PTEX150, the complete ETRAMP family (ETRAMP4, 5, 10.2, and 10.3), and MAP1, recently characterised in collaborative work (Paoletta et al. 2025, bioRxiv).

These comparisons illustrate that whilst multiple spatial proteomics approaches can provide valuable complementary data, individual predictions must be interpreted cautiously. We encourage readers to cross-reference predictions from both studies using our interactive tools.

We believe that our brief comment in the Discussion of our paper addresses the differences between these two datasets, but do not think that addressing the specific differences that have been discussed here would be beneficial for the manuscript.

<u>Comment 1.7</u>
Fig S4 is not legible at current size.

Author response: Thank you, we have now split the original Figure S4 into two figures (S4 and S5) to address this concern.

<u>Comment 1.8</u>
Discuss how these results compare with hyperLOPIT assignments published for T. gondii and Cryptosporidium in terms of methodologies and coverage.

Author response: The methodology used is near identical between the published datasets and these details are all accessible to the reader in the methods for the three studies. 'Coverage' is a more difficult measure to compare because each organism has a different number of genes, different cohorts of stage-specific expression patterns, and unknown variation in the number of proteins in the equivalent compartments (noting that some compartments have very different functions between species, such as the *Cryptosporidium* mitochondrion). We have already provided a relatively detailed general comparison of the *Plasmodium* with *Toxoplasma* hyperLOPIT maps in the discussion paragraph starting at line 450. A thorough examination of all of the similarities and differences of these rich datasets is unfeasible in the scale of this report, but these comparisons will provide many interesting lines of enquiry and follow-up analyses for future studies by the field.

Reviewer #2 (Remarks to the Author):

Overall Review:
The manuscript presents a comprehensive compartment proteomics analysis of the Plasmodium falciparum schizont stage, providing significant insights into the subcellular localization of proteins and their roles in parasite biology and evolution. The application of hyperplexed Localisation of Organelle Proteins by Isotopic Tagging (hyperLOPIT) is robust, and the results contribute valuable knowledge to the field. However, the following adjustments are requested to improve clarity and address some specific methodological concerns.

<u>Comment 2.1</u>
TMT-11plex Labeling of Samples: It is noted that each TMT experiment appears to be missing a channel that could be used for labeling a universal pooled sample. Utilizing one channel for a pooled internal standard across all experiments would greatly facilitate the normalization and quantification of data across the different experiments, thereby increasing

the robustness and comparability of the results. Clarify why one channel in the TMT-11plex was not utilized for a universal pooled sample.

Author response: Although TMT 11-plex reagents were used, only 10 channels were retained for downstream analysis for the following reasons.

First, hyperLOPIT relies on correlation-based analysis of abundance distribution profiles across gradient fractions, and not absolute quantification between experiments. The critical information is the shape of each protein's distribution pattern within an experiment, not comparison of absolute abundances across experiments. Concatenating data from multiple experiments (as we did for S1-S2 and S1-S2-S3) does not require or benefit from normalisation to a pooled standard because each experiment's profiles are independently informative.

Second, initial data generation including all 11 channels where the 11$^{th}$ represented the densest fraction of the gradient. Our preliminary analyses including this channel (TMT126) indicated that this channel introduced substantial confounding signal that degraded compartment resolution. Inspection of abundance distribution profiles and t-SNE projections showed anomalous behaviour of known marker proteins and poor separation of expected compartments. We hypothesised that this fraction was disproportionately enriched for ribosomal and proteasomal proteins arising from active protein synthesis and degradation during sample processing, contributing to apparent dual location signals and obscured genuine subcellular distribution signals. Exclusion of the TMT126 channel dramatically improved compartment resolution and marker protein clustering.

Comment 2.2

Exclusion of the S3 Sample in Figure 2 D&E: Figure 2 D&E presents results excluding the S3 sample. The exclusion of this sample might introduce biases or reduce the comprehensiveness of the data interpretation. Please provide a rationale for excluding the S3 sample in the context of those figures, perhaps adding a brief discussion of how this exclusion impacts the results and their reliability.

Author response: The decision to analyse S1-S2 separately from S1-S2-S3 was driven by the complementary nature of these datasets rather than any quality concern.

As described in the Results (lines 195-207), the S3 dataset was specifically generated from purified merozoites to resolve the rhoptry proteome (see Figure S1). Rhoptry proteins failed to cluster coherently in the S1-S2 schizont samples, likely due to dual localisation (resident in merozoites and secreted into host compartments from the previous invasion cycle). The merozoite preparation successfully resolved rhoptries as a single distinct cluster (Figure 2B), enabling classification of 44 rhoptry proteins from the S1-S2-S3 analysis.

However, the S1-S2 schizont-only dataset contains 3,000 proteins compared to 2,496 proteins in the three-way overlap (Figure 2A), providing substantially greater proteome coverage for the 23 non-rhoptry compartments. The S1-S2 analysis therefore maximises the number of detected proteins that can be assigned to these compartments. We then incorporate the rhoptry assignments from S1-S2-S3 to produce the final combined dataset of 1,646 classified proteins across all 24 compartments (Figure 2E, Table S2).

This dual-analysis strategy leverages the strengths of each dataset: S1-S2 for maximum coverage of most compartments, and S1-S2-S3 for rhoptry resolution. This approach maximises comprehensive protein assignment across the full range of subcellular niches whilst avoiding the proteome coverage loss that would result from restricting analysis to the three-way overlap.

Comment 2.3

Single Cell Proteomics: Single-cell proteomics on red blood cells (RBCs) might be crucial for this type of study because of the unique and significant biological insights it can provide. Infected RBCs can exhibit significant heterogeneity in terms of parasite load, stage of schizont development, and expression levels of both host and parasite proteins. Single-cell proteomics of an RBC could allow for the detailed analysis of these variations, providing a granular view of how the parasite interacts with the host cell at different stages of infection. Including the authors' perspective on this front in the discussion would be beneficial, as single-cell proteomics is very feasible nowadays.

Author response: Whilst single-cell proteomics could indeed provide insights into cell-to-cell heterogeneity across infected RBCs, such as variation in parasite load or developmental stage, this addresses a different question than our study. We sought to determine where proteins localise within the parasite cell (i.e., subcellular compartmentalisation), not how protein abundances vary between different infected cells. HyperLOPIT requires bulk parasite material to generate the biochemical fractionation gradients necessary for spatial mapping, making it incompatible with single-cell approaches by design.

We also note that single-cell proteomics of *Plasmodium*-infected RBCs faces substantial technical challenges that have not yet been realised in the field. The parasite occupies only a fraction of the infected erythrocyte volume, and current mass spectrometry instrumentation lacks the sensitivity to reliably measure the parasite proteome at single-cell resolution

Comment 2.4

Is this really a spatial analysis? The density gradient doesn't mean anything is being spatially visualized within the cells. Isolated compartments through this technique only gives fractionation of cells to be analyzed by TMT proteomics. Because of this, the title of the paper is slightly misleading as this is not truly a spatial technique.

Author response: We respectfully disagree with the reviewer's interpretation. The term "spatial proteomics" is well-established in the field to describe methods that determine subcellular protein localisation ie. which cellular compartment or organelle a protein resides in (PMID: 40195513). This is distinct from imaging-based spatial methods that provide positional coordinates within intact tissues or cells.

HyperLOPIT is a widely accepted spatial proteomics technique that has been extensively published and validated across multiple organisms, including apicomplexan parasites (*Toxoplasma gondii*, *Cryptosporidium parvum*) and African trypanosomes (Barylyuk et al. 2020, Guerin et al. 2023, Moloney et al. 2023). The method assigns proteins to subcellular compartments based on their physical behaviour during density gradient fractionation - proteins that share the same subcellular location co-fractionate. Machine learning then leverages these co-fractionation patterns, calibrated with marker proteins of known localisation, to predict compartment membership for unknown proteins. The result is a map of protein distribution across cellular organelles and compartments, which is precisely what spatial proteomics seeks to achieve.

Our title accurately reflects the content and methodology of our study, and the norms of the field. We determine the spatial distribution of the *P. falciparum* proteome across 24 distinct subcellular niches, providing spatial context for understanding protein function. The fact that spatial information is obtained through biochemical fractionation rather than microscopy does not make it any less spatial, rather it represents a complementary and often more comprehensive approach to mapping proteome organisation within cells.

Additional Minor Comments:
Comment 2.5
Discussion: Adding a few more references to comparable studies using isolated single-cell proteomics might strengthen the discussion on methodological parallels and distinctions. This will contextualize your findings within the broader scope of recent advances in the field.

Author response: To our knowledge, single-cell proteomics has not yet been applied to *Plasmodium* parasites. The technology remains at an early stage, and current mass spectrometry sensitivity presents a significant barrier for application to organisms as small as malaria parasites. Recent advances in single-cell spatial proteomics, including Deep Visual Proteomics (Mund et al. 2022, Nat. Biotechnol. PMID: 35590073) and emerging MS-based approaches (Guo et al. 2025, Nature PMID: 40011722) suggest such analyses may become tractable as the technology matures. But currently these methods lack the resolution required to be comparable to studies such as ours.

We would also note that single-cell proteomics and spatial proteomics address fundamentally different biological questions. Single-cell approaches characterise cell-to-cell variation in protein expression across a population, whereas hyperLOPIT determines subcellular localisation of proteins within cells. These are complementary rather than competing methodologies.

Reviewer #3 (Remarks to the Author):

I co-reviewed this manuscript with one of the reviewers who provided the listed reports. This is part of the Nature Communications initiative to facilitate training in peer review and to provide appropriate recognition for Early Career Researchers who co-review manuscripts.
